# Performance of Molecular Approaches for *Aspergillus* Detection and Azole Resistance Surveillance in Cystic Fibrosis

**DOI:** 10.3389/fmicb.2018.00531

**Published:** 2018-03-27

**Authors:** Hélène Guegan, Sylviane Chevrier, Chantal Belleguic, Eric Deneuville, Florence Robert-Gangneux, Jean-Pierre Gangneux

**Affiliations:** ^1^Laboratoire de Parasitologie-Mycologie, Centre Hospitalier Universitaire de Rennes, Rennes, France; ^2^Centre de Ressources et de Compétences de la Mucoviscidose Adulte, Centre Hospitalier Universitaire de Rennes, Rennes, France; ^3^Centre de Ressources et de Compétences de la Mucoviscidose Pédiatrique, Centre Hospitalier Universitaire de Rennes, Rennes, France; ^4^Université de Rennes 1, INSERM, Institut de Recherche en Santé, Environnement et Travail – UMR_S 1085, Rennes, France

**Keywords:** *Aspergillus*, aspergillosis, cystic fibrosis, quantitative real-time PCR, azole resistance, sputum

## Abstract

*Aspergillus fumigatus* triazole resistance is an emerging concern for treating chronically infected/colonized patients. This study sought to evaluate the performance of PCR assays to detect *Aspergillus* fungi together with azole resistance in sputum samples from cystic fibrosis (CF) patients. In total, 119 sputum samples from 87 CF patients were prospectively processed for *Aspergillus* detection by means of mycological culture and four qPCR assays, 2 in-house methods and two commercial multiplex real-time PCR assays simultaneously detecting *Aspergillus* and the most relevant *cyp51A* gene mutations (MycoGENIE^®^ and AsperGenius^®^). Azole susceptibility of *A. fumigatus* isolates was assessed using Etest^®^ method and *cyp51A* gene mutation were characterized by sequencing. The overall rate of *Aspergillus* detection with the four qPCR assays ranged from 47.9 to 57.1%, contrasting with 42/119 (35.3%) positive cultures with *A. fumigatus*. The high sensitivity of PCR on sputum could then contribute to more effective grading of *Aspergillus* disease in CF patients. Five out of 41 isolated strains (12.2%) exhibited azole-resistant MIC patterns, three of which harbored *cyp51A* mutations and only 1/3 with the sequence TR_34_/L98H. Combined with culture, PCR assay achieved high sensitivity *Aspergillus* screening in CF samples. However, *cyp51A* targeting was only moderately effective for azole resistance monitoring, while *Aspergillus* resistance remains of great concern.

## Introduction

*Aspergillus fumigatus* is responsible for severe asthma or allergic bronchopulmonary aspergillosis (ABPA) in up to 15% of cystic fibrosis (CF) patients (Stevens et al., 2003; Pihet et al., 2009), though the significance of its detection is often questioned when there are no accompanying clinical signs. *A. fumigatus* is usually susceptible to triazole antifungal drugs (with the exception of fluconazole), which have proved beneficial in treating chronic pulmonary aspergillosis (CPA). During ABPA and chronic colonization, however, their use is still controversial (Stevens et al., 2003; Pihet et al., 2009).

Over the last decade, *A. fumigatus* resistant isolates to triazole have been increasingly reported, and proven associated with a markedly higher mortality rate (van der Linden et al., 2011). This decreased susceptibility is primarily due to mutations in the *cyp51A* gene encoding lanosterol 14α-demethylase, the enzyme involved in ergosterol biosynthesis (Mellado et al., 2001; Alcazar-Fuoli and Mellado, 2013). While TR_34_/L98H and TR_46_/Y121F/T289A alterations account for the majority of azole resistance cases, a large diversity of *cyp51A* mutations has also been associated with resistance (Verweij et al., 2016). Mutations have been thought to arise during prolonged antifungal therapy or prophylaxis in individual patients, yet a number of these mutated strains were cultured from patients with no previous azole exposure. Recent data suggest that the proliferating resistance is also caused by intensive use of azole fungicides in agriculture (Verweij et al., 2016). In France, the prevalence of resistant *A. fumigatus* strains grown from sputum samples of CF patients is particularly high, ranging from 4.6 to 10.6% (Burgel et al., 2012; Morio et al., 2012). On the other hand, the current prevalence of triazole resistance in immunocompromised patients with invasive aspergillosis (IA) still remains low, estimated at ≤1% in France (Alanio et al., 2011; Guegan et al., 2018).

*In vitro* antifungal susceptibility testing is thus essential for patient management. In routine practice, the detection of azole resistance is primarily based on the *in vitro* determination of minimum inhibitory concentration (MIC) from isolates. However, the data on *A. fumigatus* resistance are scarce as susceptibility testing is not always routinely performed and sputum culture lacks sensitivity. To overcome these limitations in resistance screening, molecular methods have recently been developed to detect *A. fumigatus cyp51A* gene mutations, primarily in clinical specimens. Several nested PCR assays (van der Linden et al., 2010; Denning et al., 2011; Spiess et al., 2012, 2014) and commercial kits have previously been used, such as Aspergenius^®^ (Chong et al., 2015; White et al., 2015) and Mycogenie^®^ (Dannaoui et al., 2017) targeting both *Aspergillus* and key *cyp51A* alterations associated with azole resistance, though they have yet to be evaluated in field studies, particularly involving patients with chronic infection.

In this study, we prospectively investigated the prevalence of *Aspergillus* and the triazole resistance of *A. fumigatus* in sputum samples from CF patients using both phenotypic and molecular approaches. The efficacy of two commercial multiplex PCR assays AsperGenius^®^ (PathoNostics, Maastricht, Netherlands) and Mycogenie^®^ (Ademtech, Pessac, France) were compared with two real-time in-house *Aspergillus* assays as well as cultures. The ability of these PCR assays to detect *Aspergillus* and resistance markers was compared with culture-based susceptibility testing and subsequent *cyp51A* gene sequencing.

## Materials and Methods

### Population

Over a 6-month period, (December 2015–May 2016), all expectorated sputum samples collected from CF patients as part of their routine quarterly follow-up at the *Centre de Ressources et de Compétences de la Mucoviscidose* at Rennes University Hospital (France) were included. The samples were processed for all methods each time the minimum sample volume (≥1 ml) was available.

### Sputum Culture

On reception, 1–2 mL of sputum were digested with 1X Digest-EUR^®^, Eurobio (ratio: 1:1), and homogenized for 30 min at room temperature. Samples were divided into two aliquots to perform fungal culture and PCR assays, respectively.

For the cultures, 100 μL of pellets were inoculated in two plates of fungal media (Sabouraud dextrose agar supplemented with 0.5% chloramphenicol). One was incubated at 30°C and the other at 37°C for 7 days (Borman et al., 2010). Mold types isolated from the cultures were identified by microscope.

### DNA Extraction From Sputum Samples

We extracted 1mL of digested sputum for centrifugation, and DNA was extracted from a 200 μL pellet using the QIAamp^®^ DNA Mini Kit (Qiagen) following overnight incubation with proteinase K, according to the manufacturer’s instructions. As an extraction control, 10 μL of viral DNA (DiaControlDNA, Diagenode) were added. Elution was performed in 100 μL, and the extracted DNA was stored at -20°C until PCR testing.

### Susceptibility Testing

Each *A. fumigatus* isolate recovered from Sabouraud slants was tested as an individual isolate using itraconazole (ITC) and voriconazole (VRC) Etest^®^ strips (Biomérieux, Marcy-L’Etoile, France). MICs were determined following 48 h incubation at 37°C. Posaconazole (POS) susceptibility was also tested using Etest^®^ method, only for strains with decreased susceptibility to ITC or VRC. Strains with MIC >2 mg/L for ITC and VRC, and >0.25 mg/L for POS, were considered resistant, according to recent EUCAST breakpoints for fungi (European Committee on Antimicrobial Susceptibility Testing [EUCAST], 2017).

### In-House *Aspergillus* PCR Assays

Sputum DNA extracts were tested using two real-time “in-house” PCR assays for *Aspergillus* detection.

The first PCR assay (“Af-mito”) amplified a 196 bp-sequence of *A. fumigatus* mitochondrial gene, as previously described (F: GAAAGGTCAGGTGTTCGAGTCA; R: CATCATGAGTGGTCCGCTTTAC; 5′FAM and 3′TAMRA-labeled probe 5′-ATCCCTAAACCCGCAACCAAAGGC) (Bretagne et al., 1995).

The second PCR assay (“28S”) targeted a 67 bp-fragment of the *A. fumigatus 28S rRNA* gene, employing the primers and probe used by Challier et al. (2004) (28S-466: CTCGGAATGTATCACCTCTCGG; 28S-533: TCCTCGGTCCAGGCAGG; 28S-490: FAM-TGTCTTATAGCCGAGGGTGCAATGCG-TAMRA).

Each amplification was performed in a 25 μL final volume containing 1X TaqMan^®^ Universal PCR MasterMix, 0.5 μM of each primer, 0.2 μM of probe, 2.5 μL of mix for internal control amplification (DiaControlDNA, Diagenode), and 5 μL of sampled DNA.

Amplification was performed in the following thermal conditions: 2 min at 50°C, 10 min at 95°C, and 45 cycles of 15 s at 95°C, then 1 min at 60°C, all on a StepOne Plus^®^ instrument (Applied Biosystems).

### Commercial *Aspergillus* PCR Assays

Sputum specimens were analyzed by means of two commercial multiplex real-time PCR assays simultaneously detecting *Aspergillus* and the most relevant *cyp51A* gene mutations.

MycoGENIE^®^ (AdemTech, Pessac, France) is a quadruplex real-time PCR assay which targets *A. fumigatus* (*28SrRNA* multicopy gene) in the TR_34_ and L98H regions of the single-copy *cyp51A* gene, including an internal control to monitor for sample inhibition. Amplification was performed over 45 cycles, using an LC480 PCR device (Roche, Meylan, France). When the *A. fumigatus* target was negative and the *C*t value >35 for IC amplification, or when results contrasted with those of the in-house PCRs, the samples were retested, diluted to 1:10, 1:20, and 1:50.

AsperGenius^®^ assay (PathoNostics, Maastricht, Netherlands) provides two different real-time quadruplex amplification mixtures, one for the detection of *Aspergillus* species, and the other one for identifying prevalent resistance mutations. The species multiplex assay enables specific detection of the *A. fumigatus* complex (Af), *A. terreus*, and *Aspergillus* sp. (*Asp* sp.), by targeting the *28S rRNA* multicopy gene. Samples were retested diluted to 1:10 if the internal control *C*t was >36. The resistance multiplex assay targets the single-copy *cyp51A* gene of *A. fumigatus*, and can detect the TR_34_, L98H, Y121F, and T289A regions. Distinction between wild-type and mutant *A. fumigatus* strains was performed by melting curve analysis. The differences in fusion temperatures necessary to assign resistance were interpreted following the manufacturer’s instructions.

Amplification was performed over 45 cycles, according to the manufacturer’s instructions, on a LightCycler^®^ 480 instrument (Roche). Analysis was performed on the LightCycler^®^ 480 software, using the second derivative function. The horizontal threshold was fixed above background noise and a positive result was defined by a signal detection with a *C*t value <45 cycles.

### DNA Extraction From Resistant *A. fumigatus* Strains

DNA was extracted from *A. fumigatus* cultures a minimum of 4 days old. A conidial suspension was created in MagNA^®^ Pure Bacteria Lysis Buffer (Roche) and transferred to a MagNa^®^ Lyser Green Beads tube (Roche) for homogenization with the MagNa^®^ Lyser Instrument (Roche). We then extracted 400 μL of supernatant using the MagNa^®^ Pure Compact Nucleic Acid Isolation Kit and a MagNa^®^ Pure Extraction Instrument (Roche Diagnostics), according to the manufacturer’s instructions.

### Molecular Identification of Resistant Strains

Identification of *A. fumigatus* isolates with ITC MIC >2 mg/L was further confirmed by *beta-tubulin* gene sequencing.

PCR amplification was performed using *Bt2a* and *Bt2b* primers (Glass and Donaldson, 1995). Amplification reaction and subsequent sequencing were performed as previously described (Comacle et al., 2016). Sequencing was performed using an ABI PRISM^®^ 3130 Genetic Analyzer (Applied Biosystems). Bidirectional sequences were analyzed using Seqscape^®^ software v.2.5 and assessed within the GenBank public database using the BLAST Search program for comparison and species identification.

### *Cyp51A* Gene Typing

The whole *cyp51A* gene and its promoter were sequenced in both strands from all *A. fumigatus* strains with elevated MIC values, using five sets of primers: PA5 and PA7 (Mellado et al., 2001), AF306F and AF855R, AF766F and AF1330R, AF1179F and AF1709R, and AF1426F and AF2025R (Alanio et al., 2011).

The PCR mixture contained 5 μL of DNA extract and 20 μL of mix composed of 0.625 U of GoTaq^®^ Hot Start Polymerase (Promega), 1x Colorless GoTaq^®^Flexi Buffer (Promega), 2 mM of MgCl_2_ (Promega), 0.8mM of dNTP mix (Eurobio), and 0.2 μM of each primer. The amplification program consisted of 5 min at 94°C, 30 cycles of 30 s at 94°C, 30 s at 58°C, and 1 min at 72°C, followed by a final step of 10 min at 72°C.

Following purification and sequencing as described above, sequences of resistant strains were compared to the wild-type *A. fumigatus* sequence CM 237 (GenBank accession number AF338659), at http://blast.ncbi.nlm.nih.gov/Blast.cgi.

### Statistical Analysis

Data analysis was performed using GraphPad PRISM^®^ v.5.02 software. For continuous variables, the Mann–Whitney test was used. A *p*-value of 0.05 was considered statistically significant.

## Results

### Study Population

One hundred and nineteen specimens were collected in the context of routine clinical follow-up from 87 CF patients (sex ratio: 1.1), aged 4–59 years old (mean age: 26 ± 13 years), and included for both culture and PCR assays. Of the 87 patients, 37 (42.5%) were receiving triazole therapy at the time of sampling, specifically itraconazole (*n* = 25), posaconazole (*n* = 11), or voriconazole (*n* = 1).

### Detection of *Aspergillus* in Sputum Samples

As depicted in **Table 1**, *A. fumigatus* grew in 42 of the 119 sputum samples (35.3%), corresponding to 33 distinct patients. The two in-house Af-mito and 28S PCRs were positive in 65 (54.6%) and 68 samples (57.1%), respectively. Only 37 and 38 of the 42 sputum samples grown with *A. fumigatus* yielded positive PCR results with Af-mito and 28S PCR, respectively. Roughly similar results were recorded with the two commercial PCRs (Mycogenie^®^ and Aspergenius^®^), which amplified 64 (53.8%) and 57 (47.9%) of the total 119 samples, respectively.

**Table 1:** *Aspergillus* PCR results according to sputum culture results (*n* = 119).

Culture results	Positive *Aspergillus* PCR assay results *n* (%)				
	Af mito PCR	28S PCR	Mycogenie^®^ PCR	Aspergenius^®^ Af PCR	Aspergenius^®^ *Asp* sp. PCR
All (*n* = 119)	65 (54.6)	68 (57.1)	64 (53.8)	57 (47.9)	64 (53.8)
Positive culture for *Aspergillus*					
*A. fumigatus* (*n* = 42)	37 (88.1)	38 (90.5)	31 (73.8)	33 (78.6)	35 (83.3)
Non*-fumigatus Aspergillus* species^a^ (*n* = 5)	2^b^ (40.0)	2^b^ (40.0)	2^b^ (40.0)	2^b^ (40.0)	3^b,c^ (60.0)
Positive culture for other molds^d^ (*n* = 19)	2^e^ (10.5)	3^e^ (15.8)	3^e^ (15.8)	1^e^ (5.3)	2^f^ (10.5)
Negative culture ^g^ (*n* = 55)	26 (47.3)	27 (49.1)	30 (53.6)	23 (41.8)	26 (43.3)

*^*a*^A. nidulans (n = 1), A. versicolor (n = 1), Aspergillus sp. (n = 1), A. terreus (n = 1), A. flavus (n = 1). ^*b*^Samples grown simultaneously with A. fumigatus and flavus (n = 1), A. fumigatus and A. terreus (n = 1). ^*c*^A. nidulans (n = 1). ^*d*^Scedosporium sp. (n = 6); Penicillium sp. (n = 8); Rasamsonia argillacea (n = 2); dematiaceous molds (n = 2); unidentified mold (n = 1). ^*e*^All were Scedosporium sp.. ^*f*^Scedosporium sp. (n = 1), Penicillium (n = 1) ^*g*^Yeasts were not taken into account. PCR, polymerase chain reaction.*

Of note, the use of internal control for PCR inhibitors monitoring showed the presence of inhibitors to be prevalent in sputum samples. For in house-PCRs, 28 out of 119 (20%) samples yielded a positive signal for *Aspergillus* detection after dilutions (up to 1:50).

Interestingly, all PCRs detected *A. fumigatus* in a large number of samples that were negative in culture. As many as 41.8% to 53.6% of the 55 negative culture samples were, in fact, positive with at least one of the four PCRs. Mycogenie^®^ achieved the highest sensitivity in these samples, detecting 30/55 positive specimens (53.6%), whereas the AsperGenius^®^ PCR targeting *A. fumigatus* (Af) offered the lowest sensitivity (23/55, 41.8%). The AsperGenius^®^ *Asp* sp. assay was positive in 26/55 negative culture samples (43.3%), two of which tested positive exclusively for this target, suggesting that non-*Aspergillus* DNA was present in these samples.

None of the three sputum specimens grown with only non-*A. fumigatus* species yielded positive results with any of the four PCRs specifically targeting *A. fumigatus*. Conversely, Aspergenius^®^ *Asp* sp. correctly detected *A. nidulans*, yet was negative for the sample grown with *A. versicolor*. Finally, as observed previously by our team, all the PCRs yielded cross-reaction, with three out of six specimens detected as positive for *Scedosporium* (Guegan et al., 2018).

### Resistance Screening Using Commercial PCR Assays

As shown in **Table 2**, no *cyp51A* gene alterations were detected by Mycogenie^®^ and Aspergenius^®^ assay in the 57 and 64 sputum samples positive for *A. fumigatus*, respectively. However, Aspergenius^®^ assay correctly amplified the four regions of the *cyp51A* gene in only 20 of the 57 assessable specimens (35%). Amplification success was variable and depended on the *cyp51A* target, with detection rates ranging from 38.6% (L98H region) to 56.1% (T289A region) (**Table 2**). This finding was probably linked to the low fungal burden in samples with incomplete *cyp51A* typing, as shown by the mean *C*t values of Af target, which were significantly higher in samples with failed amplification compared to others (32.7 ± 1.6 vs. 34.1 ± 1.6, *p* = 0.005).

**Table 2:** Detection of *cyp51A* mutation using Mycogenie^®^ and Aspergenius^®^ assays.

PCR assay		Number of positive samples (%) for				
		*A. fumigatus*	TR_34_	L98H	Y121F	T289A
AsperGenius^®^	Target amplification^a^	57 (47.9)	25/57 (43.9)	22/57 (38.6)	30/57 (52.6)	32/57 (56.1)
	Detection of mutated allele^b^	NA	0/25	0/21	0/30	0/32
Mycogenie^®^	Detection of mutated allele	64 (53.8)	0/64	0/64	NA	NA

*NA, not applicable. ^*a*^Successful detection of regions carrying targeted mutations. ^*b*^Mutated cyp51A allele detection achieved by determining melting temperature values from TR_*34*_, L98H, Y121F, and T289A region amplicons.*

### Resistance Screening Using MIC Determination

**FIGURE 1:**
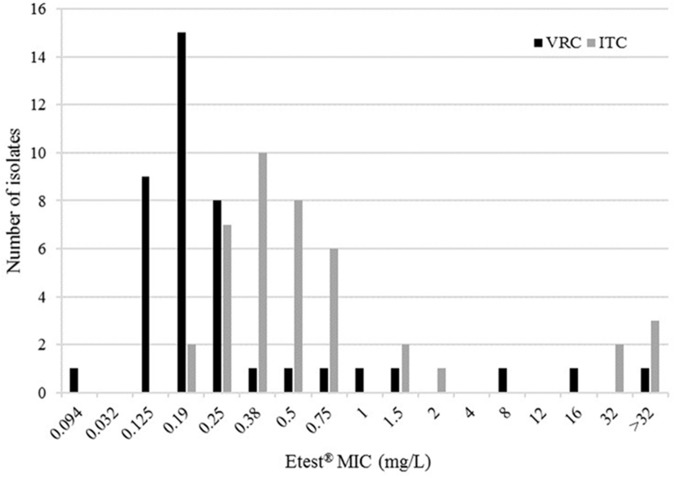
Distribution of itraconazole (ITC) and voriconazole (VRC) minimum inhibitory concentrations (MICs) of 41 *Aspergillus fumigatus* strains using Etest^®^.

We were able to assess susceptibility to triazole drugs for 41/42 *A. fumigatus* isolates. As presented in **Figure 1**, five isolates presented a resistance profile (12.2%) with high ITC MIC (≥32 mg/L). Of these five, collected from five distinct patients, three displayed cross-resistance to VRC, with high MICs ranging from 8 to >32 mg/L. Susceptibility to POS was also reduced in four of the five strains (MICs from 4 to >32 mg/L).

### *Cyp51A* Gene Sequencing of Resistant *A. fumigatus* Isolates and Occurrence During Patient Follow Up

The whole *cyp51A* gene of the five resistant isolates was further sequenced to identify the mechanism responsible for resistance. Three of the five displayed *cyp51A* gene alterations (**Table 3**). One L98H mutation (associated with a 34 bp tandem repeat in the promoter) was found in a pan-azole-resistant isolate. Surprisingly, the sputum sample from which this strain was cultured did not yield a detection of *cyp51A* mutation with any PCR assay (Mycogenie^®^, Aspergenius^®^). M220K and G54R mutations were both observed in strains with ITC and POS combined resistance.

**Table 3:** Minimum inhibitory concentration (MIC) and *cyp51A* sequencing of azole-resistant isolates.

Patient *n*°	*cyp51a* mutation (typing)		MIC Etest^®^ (mg/L)		
	Nucleotide sequence	Amino acid sequence	VRC	ITC	POS
1	None	None	16	32	0.094
2	t293a	TR_34_/L98H	>32	>32	>32
3	t659a	M220K	1.5	>32	>32
4	g160a	G54R	0.25	>32	>32
5	None	None	8	32	4

*MIC, minimum inhibitory concentration; VRC, voriconazole; ITC, itraconazole; POS, posaconazole.*

**FIGURE 2:**
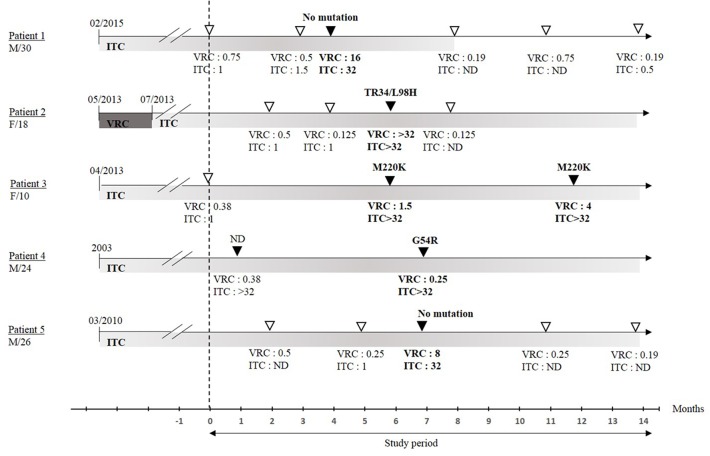
Date of treatment onset and time frame of sample collection over the study period. Isolates susceptible to azole are represented by open triangles, isolates resistant to at least one azole are represented by black triangles. Respective MIC values (mg/L) are indicated. MIC, minimum inhibitory concentration; ITC, itraconazole; VRC, voriconazole; ND, not determined; F, female; M, male; /*n*, age in years. Susceptibility testing was performed only from 09/2015.

The characteristics of patients colonized with resistant strains are depicted in **Figure 2**. All five had been receiving long-term itraconazole therapy for periods ranging from 11 months (Patient 1) to over 10 years (Patient 4) prior to sampling. No correlation was found between patient age or duration of triazole exposure and *cyp51A* genotype.

## Discussion

The burden of *Aspergillus* infections and sensitization is high among fungal diseases, particularly in the context of chronic pulmonary diseases (Gangneux et al., 2016). The emergence of triazole resistance in *A. fumigatus* has fuelled interest in molecular screening of clinical specimens. Here, we investigated prospectively whether using two commercial PCR assays was effective in (i) detecting *Aspergillus* in sputum samples from CF patients compared to culture or in-house PCRs, and (ii) in typing azole resistance.

*Aspergillus fumigatus* was the most frequent fungus isolated from CF sputum, with 35.3% of samples testing positive. PCR offered much higher sensitivity than culture, with over 40% of negative cultures yielding positive results with at least one PCR assay. There is very little data on PCR efficacy in CF sputum analyses in the literature, and our results reinforced the findings of a previous study that revealed considerable discrepancies between culture and PCR sensitivity (33% vs. 73%, respectively) (Baxter et al., 2011). We performed a standardized sputum homogenization, as this process has been shown to be crucial for these analyses (Baxter et al., 2011). The viscous nature and complex matrix of CF sputum can be responsible for inhibition and impaired detection. We thus introduced internal checks to monitor sample inhibition and systematically performed sample dilutions for in-house PCRs to minimize this phenomenon. More than 20% were found to contain PCR inhibitors. Exposure to antifungals may also contribute to limited performances of culturing but also molecular detection of *Aspergillus*. In the context of invasive aspergillosis, we previously showed the worth of molecular detection of *Aspergillus* in intensive care unit patients compared to patients that benefit from antifungal prophylaxis in hematology units (Guegan et al., 2018).

*Scedosporium* is usually considered the second most prevalent filamentous fungus in CF. In our study, we recorded a significant incidence of *Scedosporium* isolates (6/119), producing three positive PCR results. These results question an apparent lack of specificity of the *Aspergillus* PCR assay regarding *Scedosporium* species. Three isolates were sequenced using the *ITS1-5.8S-ITS2* gene. No species relationship was confirmed, as all strains were identified as part of *Scedosporium apiospermum complex* (data not shown).

While some studies have identified associations between *Aspergillus* isolation in sputum samples and the risk of pulmonary exacerbations or deteriorating respiratory function (Amin et al., 2010), the clinical relevance of PCRs in these specimens has not yet been assessed. The question remains, for example, of whether DNA fungal detection is associated with respiratory disease or is only a marker of colonization (Jones et al., 2014). In our cohort, the detection of serological markers was completed within a 3-month period surrounding sputum sampling for 26 of the 27 patients producing positive PCRs but negative cultures. *Aspergillus* antibodies or precipitins were detected in nine of them (34.6%, data not shown), indicating chronic exposure. Overall, the high sensitivity of PCR on sputum could contribute to more effective grading of *Aspergillus* disease in CF patients together with clinical signs and other biological markers, as proposed by Baxter et al. (2013).

The optimal therapeutic management of CF patients chronically colonized with *Aspergillus* is still a matter of debate. There is no consensus on the use of triazole components when *A. fumigatus* is detected in respiratory samples (Burgel et al., 2016). However, the emergence of *A. fumigatus* strains resistant to azole drugs has to be taken into account for clinical management. Here we observed reduced susceptibility to itraconazole in 5/41 *A. fumigatus* isolates (12.2%) similar to that already reported (Morio et al., 2012). The growing incidence of resistant fungi in clinical specimens is currently driving the development of new molecular approaches for rapid resistance screening directly on respiratory samples. As far as we know, this study was the first prospective evaluation of commercial multiplex PCRs to monitor triazole resistance in CF samples. These assays did not enable us to detect any hotspot mutation markers in the 57 and 64 sputum samples amplified with Aspergenius^®^ Af PCR and Mycogenie^®^ PCR, respectively. It should be noted that, in the respiratory samples from British patients with CPA, high azole resistance rates was reported using PCR (around 50%), while the cultures remained negative (Denning et al., 2011).

Globally, the sensitivity of marketed *cyp51A* PCR assays was low, primarily due to there being only a single copy of the gene. In contrast, culture isolation of resistant *Aspergillus* enabled us to sequence *cyp51A.* Of the five resistant isolates, three displayed TR_34_/L98H, M220 or G54 mutations that have already been linked to azole resistance in CF patients. Moreover, strains without any *cyp51A* alterations have been shown to be an emerging concern, suggesting the possibility there are other mechanisms responsible for triazole resistance (Rivero-Menendez et al., 2016).

Finally, PCR achieved much higher positivity in its detection of *Aspergillus* than culture, regardless of the method used (in-house or marketed). As for multiplex PCR, also able to detect *cyp51A* mutations, the presence of this gene in a single copy limits the sensitivity of a molecular approach compared to culture. There are, however, two potential limits to the resistance screening method based on MIC determination that should be discussed. Firstly, MIC is usually determined using a single colony in routine practice, yet there has been biodiversity reported within the same sputum. For example, a previous study reported up to 28% of CF patients presented complex colonization patterns containing various genotypes which succeeded each other, regardless of their antifungal susceptibility (de Valk et al., 2009). In our study, of the five patients colonized with resistant strains, only two produced several consecutive cultures growing with resistant strains (patients 3 and 4, **Figure 2**), despite no drug switches being performed between samplings. To overcome this potential inaccuracy, the use of a selective medium supplemented with antifungals for resistance detection from primary culture is currently spreading in routine practice, although there are no studies as yet reporting their relevance in CF samples (Hamprecht et al., 2017). Secondly, while there are few strains that show low MICs to itraconazole and resistance to voriconazole, itraconazole should be the preferred drug for measuring *in vitro* MIC due to the discrepancies between resistance expression against the various azoles. However, this procedure may miss some exceptional strains resistant to voriconazole despite low itraconazole MIC.

## Ethics Statement

This study was conducted in accordance with the Declaration of Helsinki and national and institutional standards. It was approved by the local ‘Rennes Ethics Committee’. No supplementary samples were drawn and investigations were considered part of routine clinical practice. However, patients or next of kin was informed of their inclusion in this study and could refuse to participate.

## Author Contributions

J-PG and FR-G designed, performed, and wrote the manuscript. HG and SC performed the analysis and wrote the manuscript. CB and ED contributed to the analysis of data.

## Conflict of Interest Statement

J-PG received a grant from Pfizer France but states that they had no influence over the research and its conclusions.The other authors declare that the research was conducted in the absence of any commercial or financial relationships that could be construed as a potential conflict of interest.
